# Label-Free Functional Imaging of Vagus Nerve Stimulation-Evoked Potentials at the Cortical Surface

**DOI:** 10.21203/rs.3.rs-4295137/v1

**Published:** 2024-05-02

**Authors:** Laura RoaFiore, Trevor Meyer, Thaissa Peixoto, Pedro Irazoqui

**Affiliations:** 1 Department of Electrical and Computer Engineering, The Johns Hopkins University, Baltimore, MD, USA; 2 Department of Biomedical Engineering, The Johns Hopkins University, Baltimore, MD, USA

**Keywords:** Vagus Nerve Stimulation, Cortical Neural Circuits, Event-related Potential, Functional Imaging, Neuromodulation

## Abstract

Vagus Nerve Stimulation (VNS) was the first FDA-approved stimulation therapy to treat patients with refractory epilepsy and remains widely used. The mechanisms behind the therapeutic effect of VNS remain unknown but are thought to involve afferent-mediated modulation to cortical circuits ^1^. In this work, we use a coherent holographic imaging system to characterize vagus nerve evoked potentials (VEPs) in the cortex in response to typical VNS stimulation paradigms, which does not require electrode placement nor any genetic, structural, or functional labels. We find that stimulation amplitude strongly modulates VEPs response magnitude (effect size 0.401), while pulse width has a moderate modulatory effect (effect size 0.127) and frequency has almost no modulatory effect (effect size 0.009) on the evoked potential magnitude. We find mild interaction between pulse width and frequency. This non-contact label-free functional imaging technique could serve as a non-invasive rapid feedback tool to quantify VEPs and could increase the efficacy of VNS in patients with refractory epilepsy.

## 1 Introduction

Epilepsy is a neurological disorder characterized by predisposition for unprovoked seizures with a high risk of recurrence. Its prevalence and burden are relatively high, with 7.60 per every 1,000 people affected over a lifetime^2,3^. While a portion of patients with epilepsy may become seizure-free through treatment with anti-epileptic drugs (AEDs), 20–40% of patients will continue to have seizures^4^. These patients have “refractory” epilepsy, and treatment options range from surgical resection of epileptogenic zones to implantation of surgical stimulation devices.

One treatment falling within the latter category is vagus nerve stimulation (VNS). VNS has been an FDA-approved treatment for medically refractory epilepsy for over two decades and its use has lately expanded to medication-resistant depression, inflammation control, metabolic syndrome, and rehabilitation aid for stroke^5^. This treatment involves the surgical implantation of a pulse generator near the patient’s clavicle and electrical leads that terminate in electrodes wrapped around the vagus nerve. The system delivers electrical impulses to the vagus nerve at programmed intervals to modulate activity of the nerve. There are many adjustable stimulation parameters defining the applied stimulation for each patient - including amplitude, pulse width and frequency - which must be decided and fine-tuned individually after implantation.

Original parameters for clinically implanted vagus nerve stimulation devices came from a study conducted on dogs in 1992^6^. These parameters were later employed in the initial clinical trials for vagus nerve stimulation in epileptic patients and later on in patients with depression^7–9^. It was later suggested that modifying these stimulation parameters could optimize the efficacy outcomes of treatment in refractory patients^10^; however, population studies across animals as well as clinical studies examining this theory were widely variable in result, making it difficult to compare and contrast parameter optimization. Additionally, some of these studies reached contradictory conclusions on effective parameter combinations - further confounding the process of parameter selection^11,12^. The dilemma facing these studies was largely the same, and arose primarily due to the difficulty in obtaining feedback on the efficacy of chosen parameters. As the clinical endpoint for VNS parameter selection is a reduction in seizure frequency, determining success requires weeks to months of waiting to determine rate of spontaneous seizure occurrence - a process that is plagued by many confounding variables which simultaneously impact seizure onset.

Unfortunately, as these previous studies underscore, optimizing stimulation parameters for individual patients remains a tedious process without any rapid-feedback tools like functional imaging protocols to improve efficacy. Manufacturer guidance suggests that clinicians begin with the most efficacious settings for their device and electrode, and make incremental changes based on observed seizure reduction in the weeks to months after each change^13^. Some studies have also used pain and perception thresholds as reference points for calibration, or other peripheral mechanisms like heart rate and heart rate variability^5,12,13^. The starting value for stimulation intensity (current amplitude) is 0.25 mA in nearly all patients. Depending on the patient’s responsiveness to this initial intensity, stimulation is then increased to a range of 1.25 – 2.00 mA over the next few months. The most prevalent stimulation settings outside of the intensity are pulse width 250 – 500 *μs*, frequency (20–30 Hz), and time cycling (30s on and 3–5 minutes off).

Prior investigations into central nervous system responses to VNS involve the use of methodologies such as functional magnetic resonance imaging (fMRI) as well as electroencephalography (EEG). However, these studies have often produced conflicting results even on the functional response to stimulation. Recent work using EEG has found that VNS stimulation at 10 Hz can produce changes to EEG power^14,15^; however, these results directly contradict findings from prior EEG studies, which found that stimulation frequencies did not produce any observable effects on EEG power^16,17^. Recent fMRI findings showed distinct differences in effects between invasive VNS and transcutaneous VNS^18^. Additionally, invasive EEG studies on acute invasive VNS have found opposing effects of similar stimulation parameters on EEG spectral and broadband power^19,20^. A more recent finding suggests that while there may be variable results across cortical networks in the stereotactic EEG of different patients, in some individuals there are distinctly similar responses with certain stimulation paradigms^14^. A possible explanation for the high variability in functional imaging results across the prior literature lies in EEG and fMRI’s lack of high enough spatiotemporal resolution to capture subtle effects of VNS on cortical circuits.

Several problems thus remain with vagus nerve stimulation: namely, there is no label-free modality with a high enough spatiotemporal resolution to visualize a patient’s VNS-evoked neural response in the cortex as required to tune their stimulus parameters for enhanced treatment efficacy. We utilize a coherent optical system to characterize the dynamics of vagus nerve-evoked potentials at the cortical surface. Visualization of the vagus nerve-evoked potentials (VEP) modulation was then recorded as a funciton of the stimulation amplitude, pulse width and frequency. Without the need for structural and functional labels like those necessary in two-photon calcium imaging, we demonstrate that vagus nerve-evoked potentials are strongly modulated by stimulation current amplitude and moderately modulated by stimulation pulse width, while there is little to no short-term modulation as a result of stimulation frequency. We find that an effect size analysis supports these conclusions, with stimulus amplitude having the largest effect size and stimulus frequency having the smallest.

The use of coherent holographic imaging to visualize the dynamics and modulation of vagus-evoked potentials at the cortical surface explored in this study is a novel, promising step towards visualizing neural responses to vagus nerve stimulation for the purpose of enabling objective, quantitative feedback of stimulation efficacy. This manuscript aims to demonstrate the validity of this functional imaging technique, and to thoroughly characterize vagus-evoked responses as they relate to the following VNS stimulation parameters: amplitude, pulse width and frequency. Success in quantifying VNS stimulation parameters in rodents will motivate future studies in translating this functional imaging technique to humans.

## 2 Methods

### 2.1 Optical

Previous studies have found evidence of optical property fluctuations in neural tissue *in-vitro* that are well-correlated to concomitant neural activation ^21–24^. These optical changes have become of high interest, as these signals represent a feasible functional imaging approach to capturing aggregate neural responses without the need for structural or functional tags, such as those in calcium imaging or fluorescence spectroscopy.

To record functional imaging correlates of VNS, we make use of a phase-driven coherent optical system called “digital holographic imaging” (DHI) developed by the Applied Physics Laboratory, to detect population-level neuronal responses for use *in-vivo*^25^. DHI is a label-free functional imaging technique which uses holography to measure nanometer-scale tissue displacements with high spatiotemporal accuracy.

Following the works presented by Lefebvre^25^, our optical imaging system uses an interferometer to illuminate the exposed cortical surface with a coherent laser source (BOA1310P, ThorLabs) at skin-safe power. The scattered light from the tissue reflects back into the optical system, where it is mixed at the focal plane array with a second beam - the reference beam, which is derived from the same coherent laser source - to create a hologram. A description of the beam path can be found in the supplementary information. An image of the cortical surface containing both phase and magnitude information is then obtained by taking the Fresnel Transform of the hologram. The phase information in these complex images is used to track phase changes from optical scatterers within the tissue to calculate the velocity of neuronal tissue displacement. This velocity profile serves as the basis for measuring aggregate cortical activation. Imaging frames recorded a roughly 4 mm diameter area of tissue at a 64×64 resolution at 4 kHz, and were triggered using custom MATLAB and Python software^25^.

In each animal, the imaging system was roughly focused on a section of cortical surface near the primary somatosensory cortex with minimal vasculature, as this area has been shown to be activated in VNS paradigms^1^. Rats were positioned and imaged on a floating table to limit artifacts from movement. After progressively increasing stimulation amplitude to observe a clear neural response, the region of interest was imaged at several depths separated by 1 mm. The depth at which the largest response occurred was selected as the principal region of interest. The largest response was typically noted at around 1–2 mm below the surface of the dura.

### 2.2 Electrical

#### 2.2.1 Twisted Pair Electrode

Twisted pair electrodes were prepared by depositing 3–4 *μm* of parylene on two 0.25 mm-diameter silver wires and twisting together. The ends of one end were cut to expose the wire tips to the recording site, and the other ends stripped and connected to an AM Systems Differential AC Amplifier (Model 1700) configured with between 60dB - 80dB of gain and a bandpass filter from 10Hz to 10kHz.

#### 2.2.2 Cuff Electrode

A custom-made cuff electrode was made by sewing 75 *μm* Platinum Iridium wire into a silicone tube (AM Systems, catalog #806700), creating a two rows of electrical contacts on the inner surface each measuring 1 mm long and separated by a 1 mm gap. The cuff’s outer surface was insulated with both a teflon coating on the wire and an additional medical-grade silicone layer.

#### 2.2.3 Stimulator Circuitry

A mirrored Howland current source, as described in ^26^, was used with the addition of larger DC blocking capacitors (labeled C2) to enable biphasic square wave stimulation. With the cathode proximal, bursts of 50 negative-positive pulse pairs were applied, cathode-first, with 200 *μs* between pulses. At least 35 seconds were reserved after each pulse burst to allow for the tissue’s return to baseline. Orofascial or neck-related musculature twitching (which can occur as off-target effects of VNS) was not observed in any of the rats included in this study. All control waveforms were generated using an NI USB-6353 DAQ at 250 kHz and custom-made scripts in MATLAB and Python.

### 2.3 Surgical

Female (*N* = *7*, 274 ± 39 g; range 218–308 g; Envigo) and male (*N* = *10*, 287 ± 27 g; range 237–324 g; Envigo) Long Evans rats were anesthetized with an intraperitoneal injection of urethane (1.4g/kg). A thermostatically-controlled heating pad maintained core body temperature at 37°C throughout the course of the experiment.

All surgical procedures were approved by the Johns Hopkins University Animal Care and Use Committee. Data from a total of 17 animals is presented, and an additional 22 animals were used in methodology development for this study. Animals were housed in same sex pairs under a 12:12: light-dark cycle, with ad libitum access to food and water. Additional animal information can be found in the supplementary information.

#### 2.3.1 Cuff Electrode Implantation

A sagittal incision of approximately 3 cm in length was made on the ventral aspect of the neck. The submaxillary gland and connective tissue were retracted. The left sternomastoid and omohyoid muscles were blunt dissected and separated until the left vagus nerve was revealed. The left vagus nerve was characterized as lying lateral to the carotid artery and was isolated from the artery and the remaining vessels in the carotid sheath. Once isolated, the nerve was sleeved into the lumen of the cuff electrode. The cuff electrode was then sutured around its circumference to secure the nerve inside and ensure appropriate electrode contact. The leads from the electrode were drawn between the subcutaneous fascia and musculature to the initial incision, which was then sutured around the leads to secure them. Following the end of imaging and recording, euthanasia (sodium pentobarbitol with phenytoin) was administered intraperitoneally.

#### 2.3.2 Venous Catheterization

The femoral vein of some study animals was cannulated in order to administer rocuronium paralytic. An incision of approximately 15 mm was made in the inguinal area and a blunt dissection of the connective tissue was performed until the femoral vein and artery were revealed. The catheter was connected to a three-way stopcock, which was connected to two syringes containing heparizined saline (20 U/mL) and rocuronium (10 mg/mL), respectively. Using micro-dissection scissors, a small cut in the vein was made at a 45 degree angle, taking care not to cut through the vein. Fine tipped forceps were fed into the incision and opened, allowing for the placement of the catheter into the vein. The catheter was fully inserted, after which all knots were tightened and the last silk piece was knotted around the venal tissue holding the catheter.

#### 2.3.3 Cranial Window

Rats were then moved into a stereotaxic frame. The scalp skin and subcutaneous fascia were resected to reveal the whole dorsal area of the skull. The periosteum was removed and a circular craniotomy with a diameter of 5 mm was made using a dental drill over an area encompassing a large region of the sensorimotor cortex. The dura was kept intact for all surgeries and sterile lactated ringer’s solution irrigation was used to maintain moisture throughout the duration of the experiments. Moistened hemostatic foam was also used during extended periods between imaging to reduce bleeding and maintain hydration.

#### 2.3.4 Paralytic and Ventilation

In a subset of experiments (*N* = *8*), some rats were paralyzed with intravenous administration of rocuronium (1 mg/kg). An endotracheal tube was used to intubate the animal and the Rovent system (Kent Scientific) was used to mechanically ventilate the animal for the duration of the experiment. Rocuronium was applied through the venous catheter with an initial bolus of 5 mg/mL and updates of 1 mg/mL every 25 minutes. Mechanical ventilation was stopped for up to 10 seconds during optical recording to reduce motion.

### 2.4 Experimental

#### 2.4.1 Electrical Validation

In a subset of experiments, optical and electrical signals were simultaneously recorded for the purpose of validating the presence and dynamics of the optical signal. The tips of the twisted pair electrode were positioned to lightly rest on the surface of the dura within the optical recording field of view (FOV). Stimulus was applied at increasing amplitudes until a stimulus-locked response was observed in both electrical and optical recordings. Low, medium, and high stimulus currents were applied to verify coordinated response changes in both recordings. Shorter pulse widths (<= 200 *μs*) were required in these experiment trials to avoid contaminating the electrical response with stimulus artifact, although the optical response does not have this limitation.

#### 2.4.2 Amplitude Characterization

First, a minimum current amplitude at which no cortical response could be recorded was found. A maximum current amplitude at which the cortical response reached a maximum value and saturated was also found. Within this range, a sequence of trials with randomized current amplitudes were delivered at constant pulse width (500 *μ*s) and frequency (13 or 15 Hz to avoid heart rate coupling), with 50 pulses per trial and at least 35 s between trials. A null trial (0 *μ*A) was randomly placed in each sequence. This sequence of amplitude sweeps was repeated for a total of three sweeps throughout the course of the experiment.

At the end of the experiments following the injection of the euthanasia compound, the cessation of diaphragmatic deflection was monitored and an additional amplitude characterization was performed for control purposes. Additionally, to control for motion artifact in a population of animals *N* = *8*, a paralytic was administered throughout the stages of experimentation. In tandem with mechanical ventilation, the responses recorded here served not only as verification that the responses were not attributable to motion, but also as a control for the responses recorded without auxiliary ventilation.

#### 2.4.3 Pulse Width Characterization

After at least one amplitude characterization, an amplitude just above threshold and below saturation was chosen. A minimum pulse width at which no cortical response could be recorded was found, and a maximum pulse width duration at which the cortical response reached a maximal magnitude and experienced saturation was also found. Within this range, a sequence of trials with randomized pulse widths was performed at a constant amplitude and frequency, with 50 pulses per trial and at least 35s between trials. A null trial (amplitude of 0 *μ*A) was randomly placed in each trial sequence. This sequence of pulse width sweeps was repeated for a total of three sweeps throughout the course of the experiment.

#### 2.4.4 Frequency Characterization

To determine vagus-evoked potential modulation as a function of stimulus frequency, a nominal literature-based range of typical frequencies utilized in VNS^12^ was created from 10 Hz to 30 Hz. Frequencies above 30 Hz were not used to avoid damaging the nerve. This range of frequencies was then used for stimulation. The stimulation was delivered in blocked trials, with 50 pulses per trial and at least 35 s between trials. A null trial (amplitude of 0 *μ*A) was randomly placed in each trial sequence.

#### 2.4.5 Effect Size Characterization

After at least one sweep of each amplitude, pulse width, and frequency characterization, a combinatorial protocol was chosen to delineate the effect sizes for each parameter. In this paradigm, low, medium, and high values for each parameter were selected. These values were then used to create an array of randomized combinations to test for the effect sizes of each parameter as well as interactions between the low and high values, low and medium values, as well as medium and high values. This entire sequence of stimulus combinations was delivered in blocked trials, with 50 pulses per trial and at least 35 s between trials. The whole trial sequence was randomized and repeated 3 times over the course of the experiment, with at least 30 minutes between each sequence. A null trial with a stimulus amplitude of 0 *μ*A was randomly placed within each sequence.

### 2.5 Data Processing

#### 2.5.1 Optical

**Figure 2.**
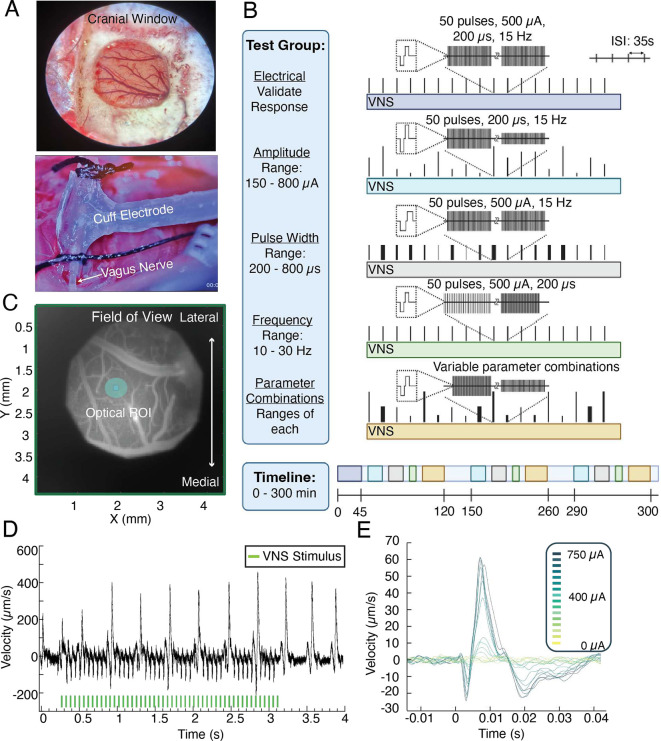
(A) Top - Representative image of a Cranial Window with vasculature. Bottom - Cuff electrode around the left vagus nerve. (B) Visualization of stimulation protocol. Five sequence types were used to consider electrical validation, amplitude response, pulse width response, frequency response, and randomized combinations of parameters. Experiments consisted of iterations of these sequences across ~300 min. (C) Hologram magnitude image showing the imaged tissue. This allows selection of ROI coordinates (teal circle) which avoided large vasculature. (D) Velocity data from a selected ROI. 50 stimulus pulses are applied (green bars), and in this trial responses can be seen after each individual stimulus pulse. (E) Overlayed responses from an amplitude sequence. Each individual trace represents the response after temporal alignment and averaging of all 50 pulses applied at that parameter setting.

The imaging data were processed using the methodology described in^25^, generating velocity measurements for the entire field of view (FOV) over time which correlated to the neural responses. To analyze response characteristics and effect sizes, a single region of interest (ROI) of a diameter of 0.4 mm was selected in a tissue area with minimal vasculature - specifically where maximal signal to noise ratio was observed (see Fig.2C). All velocity data within this ROI was averaged to mitigate phase noise from the optical system. For each trial, the individual responses to each of the 50 stimulus pulse pairs were temporally aligned and averaged, defining the evoked response. The response magnitude was characterized by the initial stimulus-aligned upward deflection in tissue velocity (see Fig.2E) which consistently occurred around 5ms ± 1ms post-stimulation. The minimum and maximum velocity along this deflection were annotated, and the difference between these points defined the response magnitude for a given trial. Any VNS-evoked response that did not have a deflection greater in magnitude than spontaneous or baseline activity was discarded.

#### 2.5.2 Experimental

Comparisons across animals for the stimulus response characteristics as well as the parameter sensitivity analyses were achieved by normalizing all response magnitudes to the maximum signal magnitude observed in a particular animal. The stimulus parameters being tested were also normalized to the maximum value of the range tested stimuli. A description of the range of parameters applied can be found in the supplementary information.

#### 2.5.3 Statistical Analyses

Data were examined for normality (Shapiro-Wilk). Non-parametric results from amplitude, pulse width, and frequency characterizations were presented as means ± standard error of the mean and analyzed using Welch’s two-tailed t-test. Effect size results were presented as standard deviations of the mean and calculated using Cohen’s D assessment. A calculated p-value of less than 0.05 was considered significantly different.

#### 2.5.4 Effect Size and Interaction

The parameter effect size, or the value of Cohen’s D, was determined by calculating the average normalized response for two parameter settings (e.g. for factor A low-high effect size, the normalized velocity response for the low amplitude setting and high amplitude settings were found separately, each including all other settings of all other parameters) and taking the difference between the means, then dividing by the standard deviation of the condition’s dataset (i.e. Low-High). (*N* = *8 animals*). The parameter comparisons were as follows: “low – high” stimulus parameters, “middle – high” stimulus parameters, and “low – middle” stimulus parameters. Interaction plots were then created for each parameter comparison to visualize the interactions of stimulus factors, where parallel lines indicate no interaction and intersecting lines signifying some interaction. Interaction plots were calculated by finding the average normalized evoked response for a condition-specific combination of parameters (i.e. A LO, A HI against P LO, P HI) and plotting these against each other to understand whether particular combinations of parameters could optimize the magnitude of the evoked signal. Each interaction plot displayed the levels of one condition on the horizontal axis and had a separate plotted line for each level of the other condition. Amplitude is represented as factor “A” or “Amp”, pulse width is represented as factor “P” or “Pulse”, and frequency is represented as factor “F” or “Freq”.

## 3 Results

### 3.1 Electrical Validation

**Figure 3.**
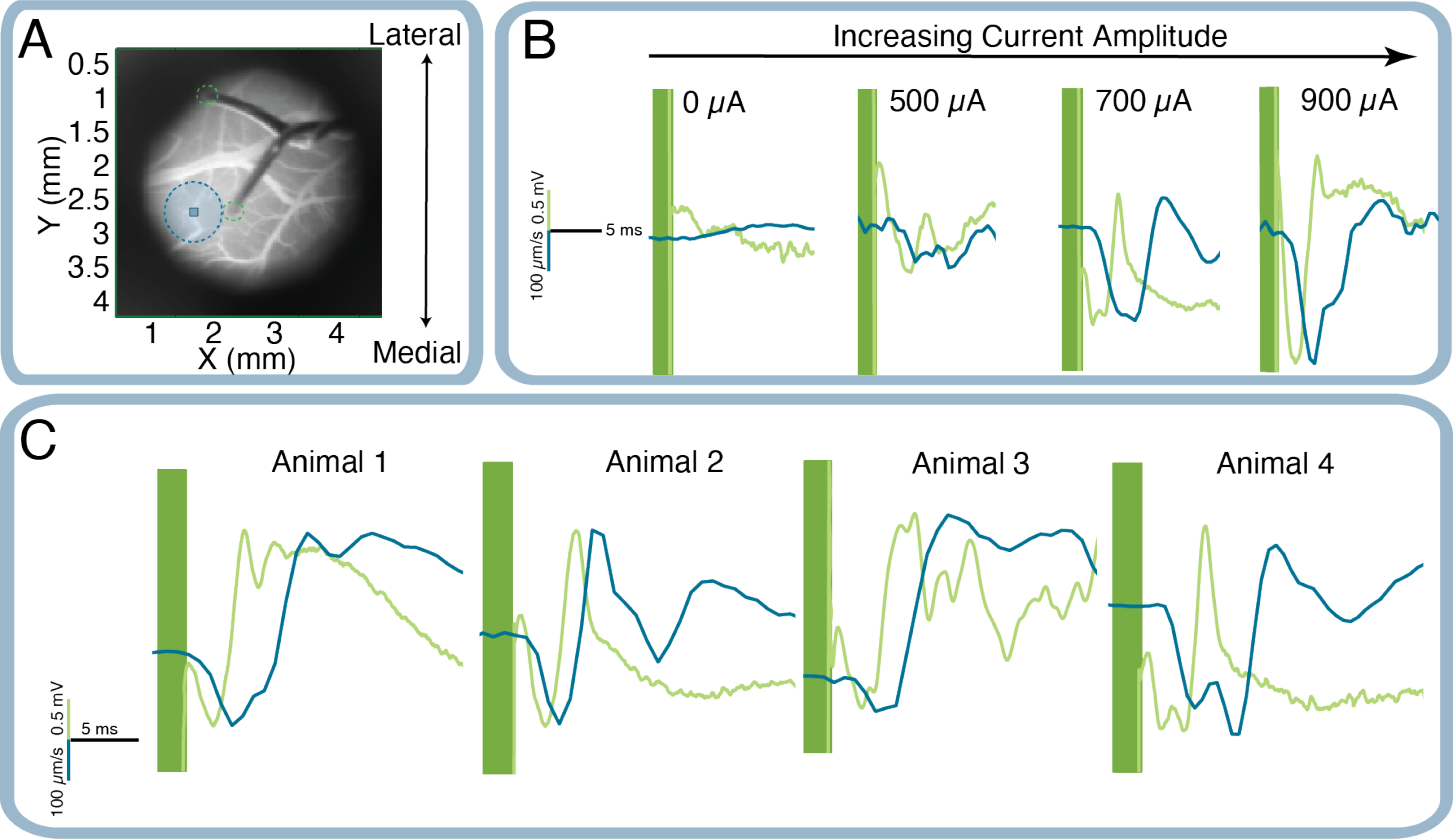
Comparison of the electrical signal (Green) with the optical signal (Blue). The stimulus artifact, present only in the electrical signal, is redacted with green vertical bars. (A) Full FOV showing the cortex with a twisted pair electrode in view. The optical ROI was placed in between the electrode tips while avoiding large vasculature, and is outlined in a blue dotted circle. The electrode tips sourcing our differential recording are outlined in a green dotted circle. (B) Representative application of stimulus with no, low, medium, and high amplitude, respectively. (C) Representative examples of responses from four animals. Although animals often had different response shapes, the rising edge considered in this study was a consistently matching feature.

The rising edge of the optical signal grows in tandem with the electrical signal (see Fig.3B), confirming a correlating dose-dependent response behavior in both the electrical and optical recordings. This protocol was confirmed in multiple animals (*N* = *4 animals*), and shape-matching, dose-dependent responses were observed between both signals. While the shape of the signal following the initial stimulus-locked response sometimes varied, there was always a correlation between the shape and magnitude of the initial upward deflection in both signals. One notable observation from Fig. 3 is that the shape of the first minimum of the signal, which is sometimes double or single peaked, consistently matches across trials and animals.

### 3.2 Amplitude Characterization

**Figure 4.**
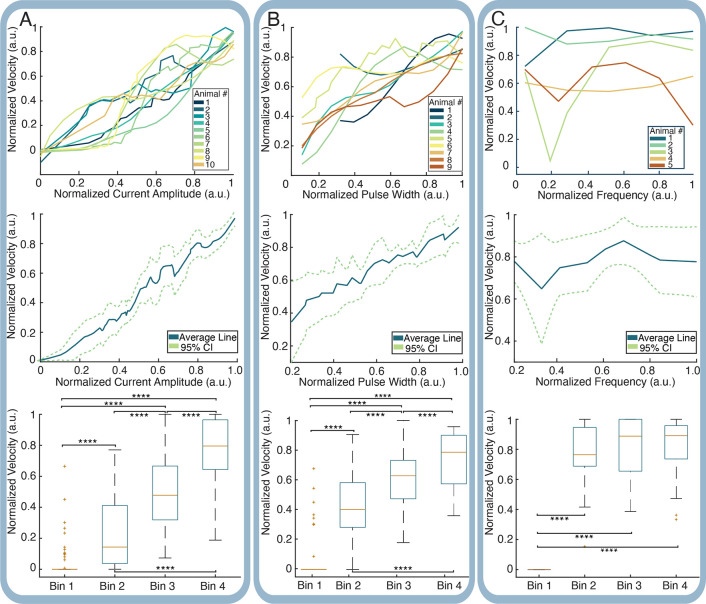
Analysis of response curves considering response magnitude related to stimulus amplitude, pulse width, and frequency. Statistical significance is indicated in relevant figures. **** = *p* < 1E-04(A) Top - Amplitude response curves from all animals. Center - Average amplitude response curve, with a 95% Confidence Interval. Bottom - Comparison of response magnitude from stimulus amplitude quartiles. (B) Top - Pulse Width response curves from all animals. Center - Average pulse width response curve, with a 95% Confidence Interval. Bottom - Comparison of response magnitude from stimulus pulse width quartiles. (C) Top - Frequency response curves from all animals. Center - Average frequency response curve, with a 95% Confidence Interval. Bottom - Comparison of response magnitude from stimulus frequency quartiles.

The amplitude characterization test demonstrated a clear increase in the response magnitude as stimulation current amplitude increased (see Fig.4A, top panel), both within and across animals (*N* = *10 animals*, 30 trials). When averaged across all trials across all animals, the resultant average input-output response to current amplitude was exponential in nature (see Fig.4A, middle panel), as is expected from neural input - output curves in prior literature^27,28^. To perform significance tests, normalized neural responses were placed into quartile bins according to the normalized amplitude tested. These bins were as follows: 0 – 0.25, 0.25 – 0.5, 0.5 – 0.75 and 0.75 – 1.0 of the maximum amplitude tested and significance between each bin was tested with Welch’s t-test (see 2.5.3 for statistical methodology). There was a high level of significance between the neural responses categorized in all quartiles. P-values between each quartile were extremely significant (see Fig.4A, bottom panel). All of these response patterns remained consistent after the application of a paralytic and after the cessation of diaphragmatic deflection following euthanasia injection. Data for these trials can be found in the supplementary information. These paralyzed and post-mortem controls, along with the electrical validation results, support that the measured response originated from stimulus-triggered aggregate neural responses and was not driven by motion artifact or other physiological correlates.

### 3.3 Pulse Width Characterization

The pulse width characterization test also demonstrated an increase in the neural response signal magnitude as stimulation pulse width increased, although it was slightly less marked than that for amplitude (see Fig.4B, top panel). This was observed both within and across animals (*N* = *9* animals, 27 trials). As with amplitude, when averaged across all trials across all animals, the resultant average input-output response to pulse width followed an upward trend (see Fig.4B, middle panel). To perform significance tests across animals, normalized neural responses were placed into quartile bins according to the normalized pulse widths of 0 – 0.25, 0.25 – 0.5, 0.5 – 0.75 and 0.75 – 1.0 of the maximum pulse width tested. There was a high level of significance between the neural responses categorized in each quartile as p-values between all quartiles were at 1.19E-05 and lower. Just as with the amplitude characterization, these response patterns remained the same in paralyzed and post-mortem trials.

### 3.4 Frequency Characterization

The frequency characterization test did not demonstrate as much of an effect on the neural response signal magnitude as frequency increased, either within animals or across animals (*N* = *5* animals, 15 trials) (see Fig.4C, top panel). When averaged across animals and trials, the neural response to frequency was minimal (see Fig.4C, middle panel), and while there was significance between the first quartile and all other quartiles (see Fig.4C, bottom panel), Welch’s t-test revealed there was no significance between other quartile relationships.

### 3.5 Parameter Effect Size and Interaction

**Figure 5.**
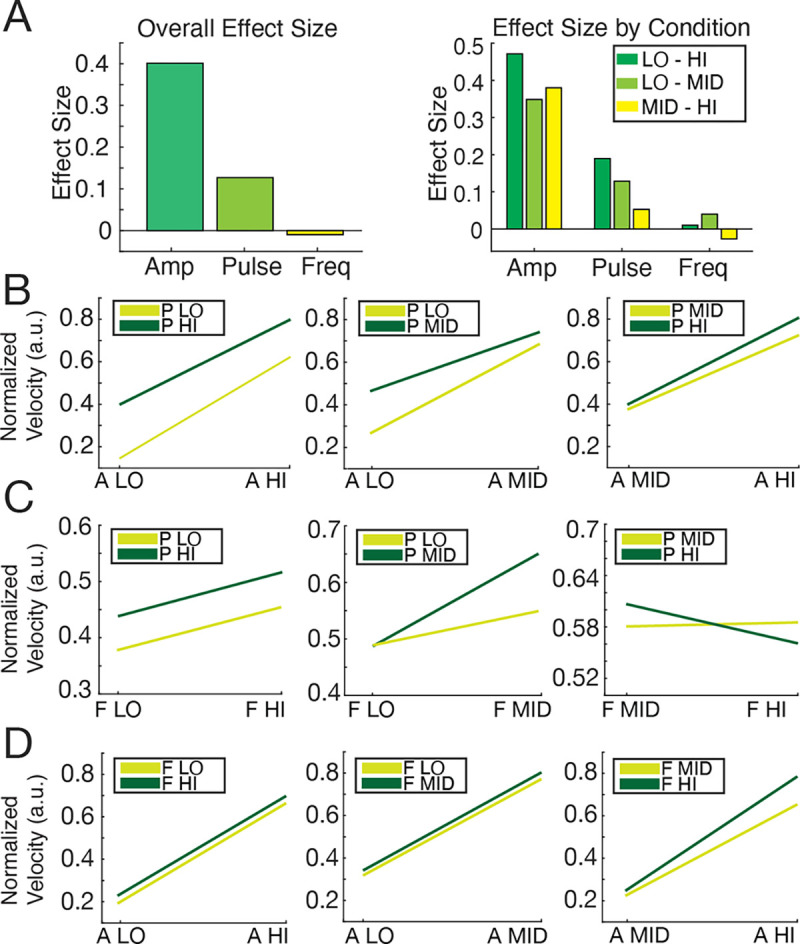
Analysis of Cohen’s D values of effect size for VEP response related to amplitude (abbreviated “Amp” or “A”), pulse width (abbreviated “Pulse” or “P”), and frequency (abbreviated “Freq” or “F”). (A) Left - Overall effect size for each parameter, showing amplitude with the greatest effect size (d=0.401), pulse width with a moderate effect size (d=0.127), and frequency with the lowest (0.009). Right - Effect size of each condition, illustrating which parameter magnitudes have the greatest effect. (B) Interaction plots for amplitude and pulse width. All plots have no intersecting points, indicating no interaction. (C) Interaction plots for frequency and pulse width. Pulse width and frequency have interactions, with P-MID and F-MID showing a larger response than P-HI F-HI and P-LO F-LO. (D) Interaction plots for amplitude and frequency. All plots have no intersecting points, indicating no interaction.

Stimulation current amplitude had the largest overall effect size (d = 0.401), which follows the above results for amplitude characterization, demonstrating that the neural response magnitude is highly responsive to increments in amplitude (see Fig.5A, left panel). The overall effect size for pulse width is smaller (d = 0.127), but still demonstrates an influence on stimulus response magnitude, which follows the results for pulse width characterization where the response magnitude was moderately responsive to increments in pulse width. Lastly the overall effect size for frequency was negative and minimal (d = −0.009), which correlates with its largely insignificant effect on the neural response magnitude (see Fig.4C) and seemingly suggests that frequency can actually inhibit the magnitude of neural response.

Effect sizes were also grouped by condition tested (i.e. low versus high parameters, low versus middle parameters, and middle versus high parameters), where the effect sizes retained the same pattern (see Fig.5A, right panel). Amplitude’s effect size is larger in all conditions, although its effect size is weaker for the mid - high parameter conditions, possibly due to response saturation. Pulse width’s effect size is moderate in the low - high and low - mid parameter conditions, although its effect size becomes much smaller in the mid - high condition. Frequency’s effect size is small but still present for the low - high and low - mid conditions, but becomes nearly negligible in the mid - high parameter condition.

There are no interactions present between amplitude and pulse width or amplitude and frequency (see Fig.5B, all panels and Fig.5D, all panels). There were interactions observed between pulse width and frequency; the interaction between pulse width and frequency in the low - mid parameters indicates an increase in neural response magnitude when mid-range frequencies are combined with mid-range pulse widths in comparison to other low - mid combinations. A more compelling interaction is observed between pulse width and frequency in the mid - high parameter interaction, where combining mid-range pulse width with a mid-range frequency gives a stronger VEP response than other mid - high combinations. There is no interaction between pulse width and frequency in the low - hi parameter condition. This implies the largest responses are elicited from middle pulse widths and middle frequencies.

## 4 Discussion

This study has used coherent holographic imaging to record the dynamics and modulation of vagus-evoked potentials at the cortical surface *in-vivo*. The results suggest that changes in stimulus amplitude initiate broad-scale modulation of aggregate neural response magnitude while changes in pulse width drive moderate modulation of neural response magnitude and changes to stimulation frequency weakly modulate or do not modulate neural response magnitude. These conclusions were supported in both isolated characterizations where all other stimulation parameters are held constant, and in effect size analysis where multiple parameter combinations were considered.

Interactions between frequency and pulse width were identified, suggesting that frequencies which are too high or too low may cause response magnitudes to break down. Upon closer inspection of individual responses, it is observed that the lower frequencies tested allowed the tissue velocity to return to a baseline between pulses, while higher frequencies initiated new responses before the previous response returned to baseline. The specific timing (frequency) and duration (pulse width) of this new activation during recovery towards baseline likely has an optimal value which may maximize response magnitude, particularly in animals where long-lasting responses were observed. These observed response timing patterns were consistent within individual animals but widely variable between animals. Overall, this study illustrates and quantifies the impact of VNS parameters on cortical response, introduces a non-contact, label-free optical recording method for probing the dynamics and modulation of these responses, and provides a foundation for future investigations into neuromodulation dynamics in other areas innervated by afferent vagal pathways.

Close inspection of the response characteristics demonstrates a delay in cortical activation from stimulus application. This delay in evoked cortical potentials is theorized to arise from synaptic signaling delays, as the initial peripheral stimulation must traverse a polysynaptic pathway through the nucleus tractus solitarius and other possible brainstem nuclei such as the locus coeruleus and dorsal raphe nuclei before reaching cortical targets^29^. As synaptic delays occur in a range of 0.5 – 1 ms and the vagal afferent pathway is polysynaptic in nature, this serves as a likely explanation for the 5 ms response delay^30^.

Prior studies have suggested that optimizing stimulus parameters can take advantage of the neural circuitry of ascending vagal afferents. One example of this is that the release of norepinephrine by the locus coeruleus is increased by vagus nerve stimulation^31,32^; however, while high amplitude and pulse width demonstrably increase the firing rate of the locus coeruleus, modulation of the stimulus frequency does not impact this firing rate^29^. The results of this study suggest that this phenomenon is also present in the cortical response to vagus nerve stimulation. While vagus-evoked potentials are modulated by amplitude and pulse width, there is little to no notable modulation by frequency. As vagus nerve stimulation changes the availability of norepinephrine in the cortex as a function of locus coeruleus firing activity, this result is perhaps to be expected.

Even in ideal acute stimulation procedures utilizing the same cuff electrode and surgical procedures across several animals, significantly different activation and saturation thresholds were found, ranging from 200 *μA* to 800 *μA*, which follows the wide variability observed in other animal studies and human applications. This variability further justifies the utility of a rapid-feedback method to individually tune stimulation parameters in each subject, which is made possible on a short time scale with the types of non-contact label-free methods demonstrated here. The findings of this study also serve as a quantitative justification for an updated parameter tuning protocol, utilizing amplitude for coarse adjustments and pulse width for finer adjustments.

The methodology developed in this study- in tandem with the coherent optical system from^25^ - to characterize evoked activity on the cortical surface are an advancement in parameter tuning capabilities compared to other functional imaging methods. This is due to the characterization of stimulation parameter effects as well as the non-contact, label-free nature of the optical system. Importantly, this optical modality avoids the presence of stimulus artifact. This is a serious limitation in electrical systems, as the short neural response delays place a limit on the testable pulse widths due to stimulus artifact. There is no other approach known to the authors that can achieve the similar spatial and temporal resolution using non-contact, label-free methodology.

## 5 Limitations

While frequency showed little to no effect on cortical activation, this may be limited by the chosen stimulation protocols. Our brief stimulus application never lasted longer than 5 seconds. This was chosen to contain enough individual responses for sufficient averaging to generate clean repeatable neural responses, while also being brief enough to enable consideration of a wide range of stimulus parameters, parameter combinations, and replicates within a reasonable experimental timeline. However, clinically effective stimulation protocols often last for a minimum of 30 seconds and can be as long as multiple hours or indefinite. Frequency may play a role in affecting circuit dynamics at these longer time scales; however this was outside the scope of this study.

The DHI system measures tissue velocity, which has been shown to correlate with neural activity. The mechanism behind this is still under investigation, and tissue motion accuracy is affected by the vascularization of the neural tissue - which experience phases of both expansion-contraction and high-low stiffness throughout the cardiac cycle. Noise from vasculature was mitigated here by choosing ROIs which avoided capturing blood vessels to maximize the signal to noise ration, preferential weighting of non-cardiac pixels, and also by choosing stimulation periods across multiple cardiac cycles to allow for averaging across multiple stimulus applications and multiple cardiac cycles. Furthermore, VNS itself can impact the cardiac cycle as its efferent projections innervate the heart. Periodically, it was observed that select frequencies near the heart rate would recruit the heart to fire in sync with stimulation. This caused cardiac artifacts to become stimulus aligned, persist through averaging, and obscure the averaged neural response. Trials containing this phenomena were obvious and were excluded from the results; however, this limited the precise frequencies available for characterization in certain animals.

Confounding motion can also be introduced by side-effects from stimulation, including muscle twitching. While we mitigated this limitation here with a verification of our findings in paralyzed animals to remove correlated muscle activity, this motion artifact remains a relevant limitation of the system that must be continuously monitored in all studies utilizing the DHI system. Other stimulation side effects, including hemodynamic effects, have also been observed in response to VNS - including blood vessel dilation - these longer time scale phenomena have been cited to occur 1 to 1.5 seconds from stimulation onset^1^. While these are stimulus locked, they do not occur on a time scale that significantly distorts the shape of the millisecond-resolution response considered in this study.

## 6 Future Work

This paper presents both a methodology and analysis tools applied to an accessible, well-established peripheral stimulation model. With this framework, implementation of these same procedures noninvasively is planned. Imaging VNS-evoked responses through skin and skull would provide a clinically translatable, direct feedback measure for VNS patients who will benefit from optimized stimulation parameters. The continued studies of VNS-evoked responses will enable a better understanding and optimization of neural activation from VNS afferent pathways in epilepsy subjects; furthermore, continued study may lead to further insights into the therapeutic mechanism behind VNS in epilepsy.

Future work will also focus on characterizing VEP spatial dynamics and may bring additional insight to the neural activation resulting from VNS - dynamics that may be more difficult to study using other functional imaging modalities due to their lack of sufficient spatio-temporal resolution. This also presents opportunities for sophisticated stimulation strategies including current steering or temporal interference stimulation techniques to preferentially impact or avoid targeted cortical areas. Preliminary data demonstrating spatially independent response dynamics and spatially targeted recruitment using current steering is shown in the supplementary information, with additional validation work ongoing. While these trials are encouraging and relevant to justify the impact of the work that our current results will support, we exclude them from the main text here as these observations justify a more focused study design and replicates to reach the statistical standards we have adhered to in our current results.

## 7 Conclusions

The combination of a novel imaging modality applied to a well-established peripheral stimulation model demonstrated the ability to characterize VNS-evoked neural responses and their dynamics in response to peripherally-driven stimulation. The results show VNS-evoked cortical responses increase as both stimulation current amplitudes and pulse widths increase. The results also demonstrate that frequency has a minimal modulatory effect on these responses. Digital holographic imaging enabled non-contact, label-free characterization and parameter tuning of VNS stimulation, enabling rapid subject-specific parameter tuning and is leading towards a non-invasive feedback mechanism and targeting strategies that could increase the efficacy of VNS in patients with refractory epilepsy.

## Figures and Tables

**Figure 1.**
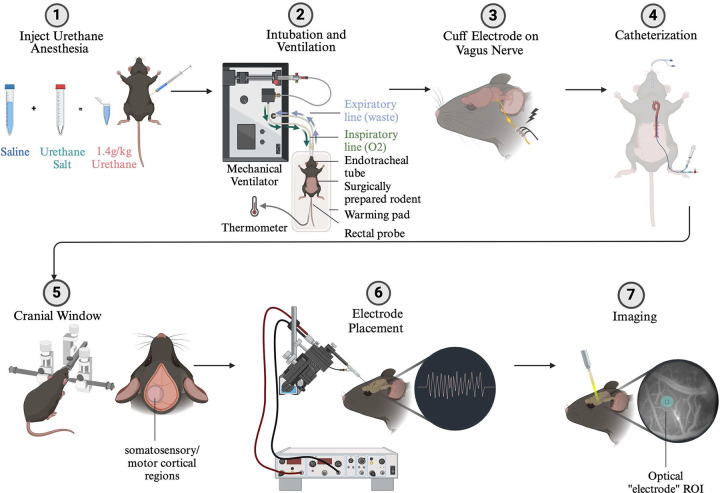
(1) Rats were anesthetized with an intraperitoneal injection of urethane. (2) Rats were intubated with an endotracheal tube and mechanically ventilated according to body weight with the Rovent system (Kent Scientific). A homeothermic system maintained body temperature throughout the experiment. (3) A custom-made cuff electrode was surgically implanted around the left vagus nerve. (4) Rats underwent a catheterization of the femoral vein in order to later introduce paralytic agent and fluids. (5) Rats were placed in a stereotaxic frame with a bite bar. A cranial window above the somatosensory and motor cortical regions was made with a dental drill. (6) Silver electrodes were placed over the exposed cortical regions using a micromanipulator (Sensapex). These electrodes were connected to a differential amplifier (AM Systems) and electrical signals were acquired (NI DAQ). (7) The frame was positioned under the beam path of the optical system (denoted by yellow beam. Color is for visual purposes and does not denote wavelength, which was 1310nm). Blood vessels were used to change the coherence length of the optical system to bring the surface of the cortex into focus and an optical region of interest was chosen (teal circle). *Figure created with* Biorender.com

## Data Availability

The datasets used and/or analysed during the current study available from the corresponding author on reasonable request.
